# *Salmonella* grows vigorously on seafood and expresses its virulence and stress genes at different temperature exposure

**DOI:** 10.1186/s12866-015-0579-1

**Published:** 2015-11-03

**Authors:** Rakesh Kumar, Tirtha K. Datta, Kuttanappilly V. Lalitha

**Affiliations:** Microbiology, Fermentation & Biotechnology Division, Central Institute of Fisheries Technology, Cochin, India; Animal Biotechnology Centre, National Dairy Research Institute, Karnal, India

**Keywords:** *Salmonella*, Seafood, Virulence factor, Stress, Gene expression qRT-PCR

## Abstract

**Background:**

Seafood is not considered the natural habitat of *Salmonella* except the river fish, but still, the incidence of *Salmonella* in seafood is in a steady rise. By extending our understanding of *Salmonella* growth dynamics and pathogenomics in seafood, we may able to improve seafood safety and offer better strategies to protect the public health. The current study was thus aimed to assess the growth and multiplication of non-typhoidal and typhoidal *Salmonella* serovars on seafood and further sought to evaluate their virulence and stress genes expression while in contact with seafood at varying temperature exposure.

**Results:**

*Salmonella enterica* Weltevreden and *Salmonella enterica* Typhi were left to grow on fish fillets at −20, 4, room temperature (RT) and 45 °C for a period of one week. Total RNA from both *Salmonella* serovars were extracted and qRT-PCR based relative gene expression approach was used to detect the expression of *rpo*E, *inv*A, *stn* and *fim*A genes at four different temperature conditions studied on incubation days 0, 1, 3, 5 and 7. *Salmonella* Weltevreden growth on seafood was increased ~4 log_10_ at RT and 45 °C, nevertheless, nearly 2 and >4 log _10_ reduction was observed in cell count stored at 4 and −20 °C on seafood, respectively. Growth pattern of *Salmonella* Typhi in seafood has shown identical pattern at RT and 45 °C, however, growth was sharply reduced at 4 and −20 °C as compared to the *Salmonella* Weltevreden. Total RNA of *Salmonella* Weltevreden was in the range from 1.3 to 17.6 μg/μl and maximum concentration was obtained at 45 °C on day 3. Similarly, RNA concentration of *Salmonella* Typhi was ranged from 1.2 to 11.8 μg/μl and maximum concentration was obtained at 45 °C on day 3. The study highlighted that expression of *inv*A and *stn* genes of *Salmonella* Weltevreden was >8-fold upregulated at RT, whereas, *fim*A gene was increasingly down regulated at room temperature. Storage of *Salmonella* Weltevreden at 45 °C on seafood resulted in an increased expression (>13 -fold) of *stn* genes on day 1 followed by down regulation on days 3, 5, and 7. Nevertheless, other genes i.e. *fim*A, *inv*A and *rpo* remained downregulated throughout the storage period. More intense upregulation was observed for *inv*A and *stn* genes of *Salmonella* Typhi at RT and 45 °C. Further, incubating *Salmonella* Weltevreden at 4 °C resulted in down regulation in the expression of *rpo*E, *inv*A and *stn* genes. Regarding *Salmonella* Typhi, *fim*A and *stn* genes were upregulated on day one, in addition, an increased expression of *fim*A was noted on day 3. At −20 °C, there was no obvious expression of target genes of *Salmonella* Weltevreden and *Salmonella* Typhi when stored along with seafood.

**Conclusion:**

Here we demonstrate that nutritional constituents and water content available in seafood has become useful growth ingredients for the proliferation of *Salmonella* in a temperature dependent manner. Although, it was absence of serovar specific growth pattern of non-typhoidal and typhoidal *Salmonella* in seafood, there was observation of diverse expression profile of stress and virulent genes in non-typhoidal and typhoidal *Salmonella* serovars. In presence of seafood, the induced expression of *Salmonella* virulent genes at ambient temperature is most likely to be impacted by increased risk of seafood borne illness associated with *Salmonella*.

## Background

*Salmonella* serovars are leading food-borne pathogens and commonly isolated from meat and poultry. More recently, presence of *Salmonella* has been reported in fish and seafood [1, 2]. Numerous reports are available on seafood implicated in the outbreak of human salmonellosis [3, 4]. Generally, animals, birds and humans are the natural host of *Salmonella*. More than 90 % of food-borne outbreaks are due to non-typhoidal *Salmonella* serovars and typhoidal group is not frequently contaminated with *Salmonella*. Despite the fact that seafood is not considered the natural host for *Salmonella* and further, it is always transported at low temperature, still, the incidences of *Salmonella* in seafood is in increasing order [5, 6]. It is reasonably well understood that the phenomenon of growth and multiplication of *Salmonella* in food environment is primarily dependent on factors like temperature, pH, availability of essential nutrients, contact surface and water activity of the food matrix. Seafood provides repertoire of elements like vital nutrients, appropriate salts and provide large amount of water to support the growth of food- borne bacterial pathogens. Survival and detection of *Salmonella* in seafood even after prolonged frozen condition is always a matter of concern for seafood consumers, processors and researchers. In case of contamination, it must be intriguing to know the ability of *Salmonella* to grow in seafood. Although, attempts have been made to understand the growth dynamics of *Salmonella* in beef, pork and chicken [7, 8], only few reports are available on multiplication of *Salmonella* in seafood.

*Salmonella* survival and multiplication in food and water environment are mainly due to its ability to respond effectively by suitable changes in gene expression pattern responsible for environmental persistence [9]. Besides an immediate cellular adaptation to stress, organisms can resist such challenges through certain changes in their genetic material like the phenomenon of gene duplication [10]. Cellular adaptation mechanism of the organism depends upon modification of certain aspects of cell physiology and supported by decrease in a ratio of unsaturated to saturated fatty acid of membrane lipid composition by intracellular signalling networks [11]. Ribosomal-RNA constitutes 82–90 % of total RNA pool in bacteria and represents the active fraction of the cellular activity and metabolic state of bacteria in the environmental samples. In the past, rRNA analysis has been used to quantify bacterial population growth rate in a mixed microflora [12]. Based on this, we hypothesize that determination of total RNA may qualitatively indicate that cells are in very active and growing mode or just present in a dormant and dying state.

Presence of various genes in bacteria is responsible for their ability to multiply and survival in food environment. Major genes involved in cell wall structural and functional integrity, and nucleic acid and amino acid metabolism are important for *Salmonella* to persist in food and other environments [13]. *Salmonella rpo* genes are mainly responsible to cope up with various environmental stresses, while *rpo*E and *rpo*H genes have been associated with thermal related stress in *Salmonella* [14]. The virulence factors and level of pathogenicity among the non-typhoid and typhoid *Salmonella* serovars has been observed to be diverse which ultimately determine the nature and disease outbreak ability of the strain to humans. An initial evaluation must be carried out to know the expression of non-typhoidal and typhoidal *Salmonella* virulent genes in contact with seafood and further, enables us to understand the level of pathogenicity outside of the host environment and their preparedness and capacity to cause infection. Role of *inv*A gene in *Salmonella* pathogenicity is well understood and this gene contributes significantly to virulence factor of *Salmonella* pathogenicity Island (SPI). The virulence factor due to *inv*A gene is reported to be responsible for invasion of gut epithelial tissue in human and animals and *Salmonella* enterotoxin (*stn*) gene has been associated with pathogenicity in *Salmonella* serovars [15]. The *fim*A gene encodes the major structural subunit of type I fimbrial protein, while this gene has been implicated in *Salmonella* pathogenicity [16]. Involvement of active role of *Salmonella* virulence genes such as *inv*, *stn* and *fim* in pathogenicity were ascertained based on in-vivo and in-vitro challenge studies and confirmed the release of specific protein or toxin molecules [17]. Although, previously expression of these genes are confirmed using gene cloning approach, perhaps now by targeting mRNA may provide good and alternative method to understand the gene expression in pathogenic bacteria. Here, we made an attempt to evaluate the expression of *Salmonella* stress and virulence genes in seafood at different temperature exposures.

## Results

### *Salmonella* growth in seafood at different temperatures

**Fig. 1:**
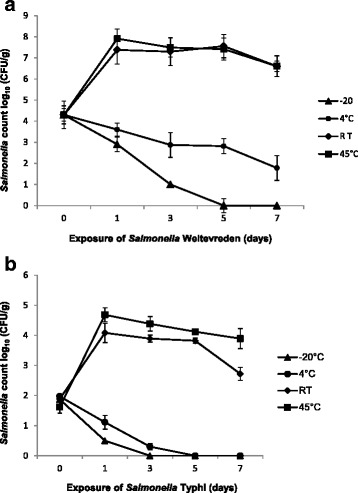
(**a**)* Salmonella* Weltevreden (**b**) *Salmonella* Typhi counts obtained on XLD plates and ChROMagar™ *Salmonella* from seafood following 1, 3, 5, 7 days of incubation at -20, 4, RT and 45 ºC. Results shown represent the mean of three independent trials with average count on XLD and ChROMagar™ *Salmonella* media. Error bars shown represent the standard deviations from triplicate replicates of each sample

Recovery of *Salmonella* Weltevreden and Typhi in seafood was determined on 0,1,3,5 and 7 days by using the agar plating method of xylose lysine deoxycholate (XLD) and ChROMagar™ *Salmonella* media. Regarding the growth of *Salmonella* Weltevreden, we observed that cell count increased from 4 log_10_ to 7 and 8 log_10_/g, at RT and 45 °C, respectively on day 1 and thereafter, cells maintained a plateau till 5^th^ day, finally population was decreased by 1 log_10_ on day 7. At 4 °C, *Salmonella* Weltevreden population followed a continual reduction pattern from 4 log_10_ to 1 log_10_. However, *Salmonella* Weltevreden reduction was much sharper in case of temperature exposure at −20 °C. In this case, initial population of 4 log _10_ CFU/g decreased to < 1 log_10_ on day 5, while on the 7^th^ day, cell counts were below the detection limit of the plate count method (Fig. 1a). Growth of *Salmonella* Typhi in seafood at different temperatures storage, cell count was increased from 1 log_10_ to 4 log_10_ both at RT and 45 °C on day one thereafter continual reduction in cell count was observed till day seven. Nevertheless, storing seafood at 4 °C has shown reduction in count of *Salmonella* Typhi from 1.8 log_10_ on day one to < 1 log_10_ on day 5 and further incubation did not yield culturable *Salmonella* Typhi. At −20 °C, we could not detect viable *Salmonella* Typhi on day 5 and 7 from the seafood inoculated with initial cell count of 3 log_10_/g (Fig. 1b).

### *Salmonella* RNA quantification on seafood

**Fig. 2:**
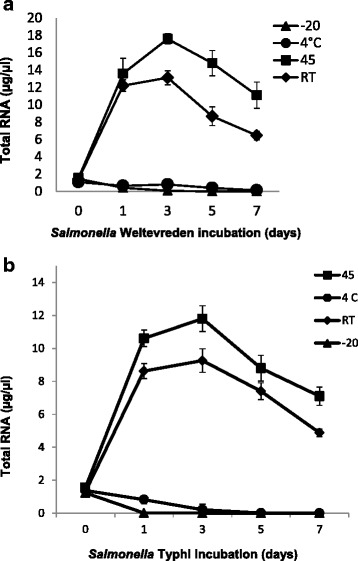
Detection of RNA from (**a**) *Salmonella* Weltevreden and (**b**) *Salmonella* Typhi following 1, 3,5,7 days of incubation given to seafood at -20, 4, RT and 45 ºC. Results shown represent the mean of three independent trials. Error bars shown represent the standard deviations from triplicate replicates of each sample

**Fig. 3:**
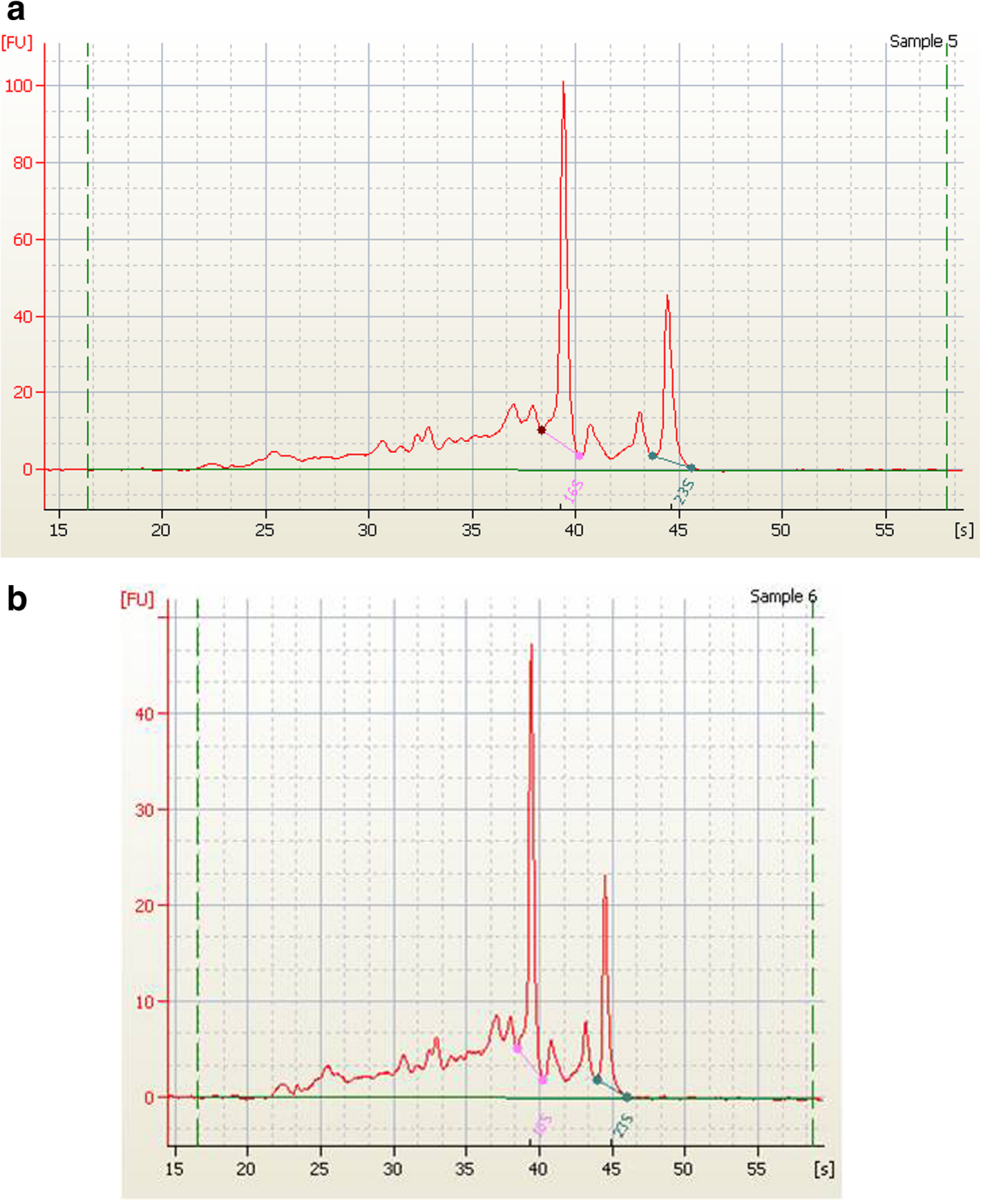
Representative sample of (**a**) *Salmonella* Weltevreden and (**b**) *Salmonella* Typhi, showing quality and integrity of 16S and 23S RNA in Fluorescence Unit (FU)

We have quantified total RNA concentration from *Salmonella* Weltevreden and *Salmonella* Typhi of the individual temperature groups. Regarding *Salmonella* Weltevreden, RNA was in the range from 1.3 to 13.11 μg/μl at RT and at RT and at 45 °C concentration was found to be increased from 1.4 to 17.6 μg/μl. However, storage at 4 °C RNA has shown considerable decrease in RNA concentrations from 1.2 to 0.14 μg/μl. Similarly, RNA concentration was an about of 7.8 ng/μl and 3.8 ng/μl on 1 and 3 day, respectively at −20 °C, thereafter, RNA was not detected on 5 and 7^th^ day (Fig. 2a). Regarding *Salmonella* Typhi, RNA concentrations obtained were in the range from 1.2 to 9.2 μg/μl and 1.2 to 11.8 μg/μl at RT and 45 °C, respectively. We observed that the total RNA concentration in the range from 5 ng to 1.2 μg/μl at 4 °C storage (Fig. 2b). Detection of RNA concentration was 14 ng/μl at −20 °C on day one and subsequently, no RNA was detected on 3, 5 and 7 day. The quality of the RNA was excellent in nature and clear pattern of 16S and 23S RNA peaks were observed from *Salmonella* Weltevreden and *Salmonella* Typhi on Bioanalyser (Fig. 3a, b). RNA integrity number (RIN) values of 7.1 were only considered for the gene expression study.

### qRT-PCR validation and reference gene

Endogenous reference gene (*gapdh*) was validated for different temperature exposures and it was found that *gapdh* was consistent and expressed uniformly across the exposure temperature. The threshold Ct values were falling in the range from 16.39 to 21.75. The temperature exposures did not show significance difference (*p* > 0.05) in Ct values. qRT-PCR amplification during gene expression was confirmed by melting curve analysis of the gene amplicons (data not shown). All primers demonstrated single peak in the melting curve graph and Tm values of *Salmonella* Weltevreden and *Salmonella* Typhi for *rpo*E, *fim*A, *inv*A and *stn* genes were 84.3, 73.5, 82.5, and 78.5 °C ±1 °C. There was no amplified product seen from NRTC which confirmed the absence of genomic DNA during qRT-PCR expression assays.

### Relative gene expression

**Fig. 4:**
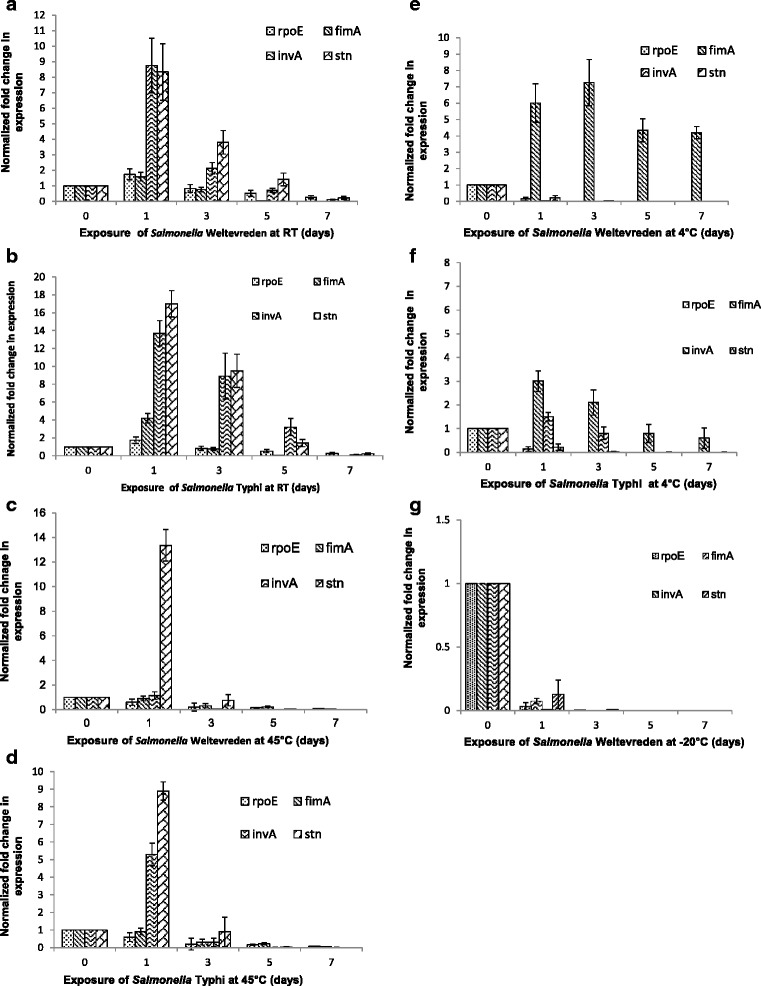
*Salmonella* Weltevreden (**a, c, e & g**) and *Salmonella* Typhi (**b, d & f**) *invA, stn, fimA,* and *rpoE* gene expression at RT, 45, 4, -20 °C over a 7 days exposure in seafood. Normalized gene expression values against housekeeping gene (*gapdh*) are shown and error bars represent the standard deviations from triplicate replicates of each sample

Differential expression of *rpo*E, *inv*A, *stn* and *fim*A genes of *Salmonella* Weltevreden and *Salmonella* Typhi during their exposure in seafood at −20, 4, RT and 45 °C were analyzed (Fig. 4). Exposure of *Salmonella* Weltevreden in seafood at RT triggered almost 8 fold upregulation in *inv*A and *stn* genes on the 1^st^ day, whereas 2 and 4-fold increase was observed for them on 3^rd^ and 5^th^ day, respectively and considerable down-regulation was observed on 7^th^ day. The *fim*A gene was increasingly down regulated throughout at room temperature except on day one. *Salmonella* incubation at 4 °C resulted in down regulation of *rpo*E, *inv*A and *stn* genes throughout exposure period from day one to seven. However, there was 6-fold up regulation in *fimA* gene expression on day one, thereafter 7.4, 4.5, and 4-fold increased up regulation in *fim*A gene was observed on 3,5,7^th^ day, respectively. We demonstrate that during the incubation at 45 °C, there was 13-fold increase in *stn gene* expression on day one and subsequently down regulation was observed on 3, 5 and 7^th^ day. Expression of *rpo*E, *inv*A and *fim*A genes was more than 10-fold down regulated on day 7 following the incubation at 45 °C. Further, at −20 °C, there was 10-fold down regulation for *rpo*E, *fim*A and *stn* on day one, further no noticeable expression was observed for all the target genes. Regarding the expression of *Salmonella* Typhi at RT, there was 13.7 and 17-fold upregulation in *inv*A and *stn* genes, respectively, on day one and 8.9 and 9.1-fold upregulated expression for them on day 3. In addition, both the *rpo*E and *fim*A genes were also found to be 1.7 and 4.2-fold upregulated, respectively at RT. Exposure of *Salmonella* Typhi at 45 °C, we report that there was 5.3 and 8.9-fold upregulation in *inv*A and *stn* genes expression, respectively, whereas, *fim*A and *rpo*E genes were observed to be down regulated throughout the storage period. Furthermore, there was 3 and 1.5-fold upregulated expression of *inv*A and *fim*A genes, respectively of *Salmonella* Typhi at 4 °C on day one. We could not get noticeable expression pattern of target genes of *Salmonella* Typhi at −20 °C.

## Discussion

Seafood is ideally considered to be free from *Salmonella* and occurrence of *Salmonella* in seafood is mainly due to cross-contamination linked with zoonotic and anthropogenic activities towards the coast lines [18]. Previously, our group has reported the widespread prevalence of *Salmonella* serovars in tropical seafood [1]. From the viewpoint of present increase in incidences of *Salmonella* in seafood, it is quite apparent that *Salmonella* remains viable and active for longer time in seafood environment. Considering the frequent detection of *Salmonella* in seafood, the present study was undertaken to assess the growth dynamics of *Salmonella* in seafood. Seafood is rarely stored at elevated temperature, however, during the post-harvest handling and transporting, it is well known that temperature abuse can result in multiplication of pathogenic bacteria. It has been seen from our results that *Salmonella* comfortably grows and multiplies in seafood at room temperature and above. Although, there was a gradual reduction in *Salmonella* load for couple of days at 4 °C, and further reduction was much sharper at later stages of storage (5–7 days). As expected, sharper decline in *Salmonella* population was observed at - 20 °C and no culturable *Salmonella* was detected on the 7^th^ day of storage. The possible reason for sharp reduction in *Salmonella* count could be due to the freezing and sometimes partial thawing step involved while withdrawing seafood samples. It is also true that reduction in *Salmonella* population at low temperature was partially due to the non-recovery of metabolically injured cells by direct plating method. The process of freezing has been reported to give detrimental effect on bacterial cell wall, resulting in faster cell death. Contrary to our study, *Salmonella* in frozen seafood without involvement of thawing step was reported to survive for more than 8 weeks [19]. Further, we could detect more than 3 and ~4 log cycle increase cell count for both serovars within 24 h of initial storage at RT and 45 °C, respectively. Similarly, a study elsewhere has reported to increase *Salmonella* Enteritidis count by 3 log cycle in pork meat kept at 10 °C for 5 days [7]. Quite contrary, there was no *Salmonella* growth reported to detect in frozen whole chicken and ground beef kept for thawing at 22 and 30 °C for 9 h [8]. Regarding the growth pattern of non-typhoidal and typhoidal *Salmonella* serovars, the study highlights that there was no inter-serovar difference in growth pattern at the ambient temperature, however, the only variation observed in the study that *Salmonella* Typhi was sharply reduced to nil at 4 and - 20 °C. This prompt reduction in cell count and low temperature sensitivity of *Salmonella* Typhi could be due to its possible adaptation to humans, the only known host. Although, proved many times earlier, we reiterate that the refrigeration and subzero temperature were found to be critical for regulating the growth of *Salmonella* on seafood. The faster *Salmonella* growth rate has been seen in seafood kept at ambient temperatures in the current investigation. This must be attributed due to the intrinsic factors like suitable nutrient composition, pH and availability of higher water content in seafood. The proximate composition of seafood is well documented and seafood is reported to be source of rare and vital nutritional elements like minerals, vitamins, lipids and amino acids that apparently support the bacterial growth [20]. Presence of such vital and ideal nutritional elements in seafood must have given impetus to the growth of *Salmonella* in seafood. In addition, contact surface and water content available in seafood are also considered vital components for growth of bacteria. We demonstrate that non-typhoidal and typhoidal *Salmonella* serovars multiplied very efficiently in seafood without further addition of external water, which, in turn, suggests that available water content in seafood is adequate for proper multiplication of *Salmonella*. The average water content in common seafood is reported to be 80 % of the body weight [20], which is rather high as compared to any other food including fresh meat. Our data supports that the inherent moisture content may have contributed to the rapid proliferation of pathogen on seafood. In addition, non- availability of competing microflora might have given the contributory effect on exuberant growth of *Salmonella* on seafood in our study. We highlight that *Salmonella* has the ability to grow seafood alone and the consequence of expedite growth of *Salmonella* in seafood alone can be serious. Taken together, we may imply that seafood has provided all necessary nutritional inputs, sufficient amount water and overall suitable environment for the growth of both non- typhoidal and typhoidal *Salmonella* serovars at favourable temperature, thus, makes seafood the most vulnerable food for the growth of *Salmonella* at ambient temperature. Further, we sought to gain insight into the dynamics of cellular activity and multiplication on the amount and content of stable RNA which ultimately indicate the well being of cellular machinery of an organism. The mRNA shows the gene expression process and overall turnover rate of cellular activity of a cell. Even in the past, r-RNA has been reported to use as an indicator of the microbial activity [21]. Similarly, we tried to establish that r-RNA can be a useful and qualitative indicator of bacterial metabolic activity when it constitutes more than 90 % of the bacterial total RNA. Quantification of total RNA was probably an effort made in this study to speculate it as a qualitative indicator of cellular growth and activity. Here, we demonstrate that quantity of RNA was proportionately related to the temperature exposure given to the organism in presence of seafood. Detection of higher concentration of RNA was obtained from *Salmonella* serovars kept at RT and 45 °C as compared to the 4 and −20 °C. We demonstrate that total RNA steadily increased upto 3 day of incubation *in* seafood, even though, there was decline in *Salmonella* count beyond day 1 on seafood following the incubation at RT and 45 °C. This highlights the existence of negative correlation between RNA concentration and cell count during day 1 to 3 in both strains. The continual progress in total RNA concentration upto day 3 even when cell count was found to be declined at same stage has indicated that seafood may either providing protective environment or prolonging the cellular activity of *Salmonella*, consequently, RNA content remained stable and active for longer time at the ambient temperature. No such phenomenon was observed for *Salmonella* stored at low temperature. It has been documented previously that starvation gives most detrimental effect on degradation of the stable RNA in bacteria and at very low growth rates as much as 70 % of the newly synthesized rRNA does not accumulate in ribosomes and apparently undergo degradation [22]. Further, results highlight that less cellular activity was occurring at 4 °C and no metabolic activity prevailed at −20 °C, could be attributed due to the frozen conditions of cell contents as well as cell death. We next tried to understand the level of expression of *Salmonella* virulence and stress gene on seafood following a diverse temperature exposure regimen. The amount of total RNA obtained from different temperature exposure groups of *Salmonella* Weltevreden and *Salmonella* Typhi on seafood are used to detect the expression of target *rpo*E, *inv*A, *stn and fim*A genes. Regarding *Salmonella* Weltevreden virulent and stress genes expression, present data revealed that *inv*A gene expressed differently at RT, 4 and 45 °C; it was substantially upregulated at RT and significantly down regulated at 4 and 45 °C (*p* < 0.05). Similarly, expression of *stn* gene of *Salmonella* Weltevreden at RT remained upregulated on day 1 and 3, and thereafter down regulation was observed on day 5 and 7. Further, we found that *stn* gene of *Salmonella* Weltevreden remained down regulated at 4 and 45 °C, nevertheless, upregulation was noted for *Salmonella* Typhi following the storage at RT and 45 °C. This signifies the induction of virulent genes in *Salmonella* Typhi with wide range of temperature in seafood. We further demonstrate that storage of *Salmonella* Typhi in seafood at RT has shown much increased (>13-fold) in *fim*A and *stn* gene expressions on day 1 and their expression pattern remained upregulated till day 5 (*p* < 0.05). We demonstrate that except *fim*A gene, the increase in expression of virulence genes *inv*A and *stn* of *Salmonella* Weltevreden and *Salmonella* Typhi primarily express at the ambient temperature in seafood. The current study demonstrated that there was apparent difference in expression pattern of virulent genes in non-typhoidal viz-a-viz. typhoidal serovar signifies the existence of higher level of virulence factors in *Salmonella* Typhi in seafood which in turn is capable of contributing real-time more vigour towards its pathogenicity as compared to *Salmonella* Weltevreden. It was previously reported that expression of *inv*A gene remained static after starvation in seawater for 3 years at room temperature [23]. Concurrently, report has shown that environmental factors such as osmolarity and temperature have crucial role for expression of *inv* genes due to DNA super coiling and reduction in linking number of DNA [24]. Based on our data, it is also intriguing to report that the amount of total RNA of *Salmonella* Typhi was much lower as compared to *Salmonella* Weltevreden, but the expression of *Salmonella* Typhi, *inv*A, *stn* and *fim*A genes were relatively high at ambient temperature. This could be due to the mRNA transcripts of *Salmonella* Typhi must be much higher in total RNA as compared to that of *Salmonella* Weltevreden.

Among the other virulence gene investigated, transcription of *fim*A gene of *Salmonella* Weltevreden was up-regulated during storage at 4 °C and significantly down regulated when stored at RT and 45 °C (*p* < 0.05). Similar observation was noted for *Salmonella* Typhi. It was reported that an 11-fold increase in activity of *fim* A promoter when growth temperature declined from 39 to 34 °C in *Porphyromonas gingivalis* [25]. A complex molecular mechanism has been proposed for the temperature controlled fimbrial circuit switch in uropathogenic *E.coli* [26]. The rate of transcription of *fim*A in *E.coli* was reported to be consistently higher at 30 °C than to 37 °C [27]. More recently, it is reported that virulence factors are regulated by temperature-sensing RNA sequences, known as RNA thermometers (RNATs) which are present in their mRNAs [28]. Taken together, our data demonstrate that *fim*A gene of *Salmonella* has an ability to induce the transcriptional mechanism even at very low temperature (4 °C). Expression of *rpo*E gene of *Salmonella* Weltevreden and *Salmonell*a Typhi in seafood remained down regulated at −20, 4, RT and 45 °C (*p* > 0.05) and no specific pattern of expression was observed for *rpo*E. The reason behind this static down regulation in *rpo*E gene could be due to its induction under the carbon starvation and osmotic stress conditions unlike this study [14]. It has been reported that *Salmonella rpo*E is not essential for its viability at high temperature. The *rpo* genes are generally expressed in stress conditions and *rpo*E and *rpo*H has been reported to involve in antioxidant defence by enhancing expression of *rpo*S in *Salmonella*.

## Conclusions

This work provides the evidence that considerable increase in *Salmonella* population takes place within 24 h and seafood can be a suitable growth medium for multiplication of *Salmonella* at ambient and above RT upto 45 °C. The temperature range for the growth of *Salmonella* spp. is 5.2–46.2 °C, where the optimal temperature range lies in between 35 and 43 °C [29]. Exposure to low temperature, typhoidal *Salmonella* was found to be more sensitive as compared non-typhoidal serovar. We provided the evidence that concentration of *Salmonella* total RNA indicates its preparedness in the form of metabolic and cellular activities to cope with environmental stress while in contact with seafood. Relative expression of stress and virulent genes of *Salmonella* reveals both in terms of activation and repression of target genes in diverse expression modes depending upon the exposure of temperature and cellular activity. Interestingly, *Salmonella* Typhi seems to be more potent and showed increased ability to induce the expression of *inv*A and *stn* genes. Expression of *fim*A gene was induced at low temperature in both typhoidal and non-typhoidal *Salmonella* serovars. It is therefore, important to point out that room temperature has been found the most ideal temperature for increased expression of virulent *inv*A and *stn* genes which signify the level of pathogenicity of organism remained high and active in seafood.

## Methods

### *Salmonella* cultures and inocula preparation

Two representative, non-typhoidal and typhoidal *Salmonella* serovars i.e. *Salmonella enteric* serovar Weltevreden and *Salmonella* Typhi isolated previously from seafood were included in this study [1]. Frozen stock of *Salmonella* Weltevreden and *Salmonella* Typhi (−80 °C) was cultured in Brain Heart Infusion (BHI) broth. The cultures from BHI broth was put onto BHI agar and single colony of *Salmonella* Weltevreden and *Salmonella* Typhi from BHI agar was streaked onto BHI agar slants. The inoculation culture was prepared by transferring culture from agar slant to BHI broth (5 ml) and one ml of overnight culture was centrifuged at 7000 × g for 2 min to settle down the cells. The pellets of *Salmonella* Weltevreden and *Salmonella* Typhi were diluted in sterile normal saline to get approximately 2x10^7^ CFU/ml and 2x10^6^ CFU/ml count, respectively. Finally, the pellets were resuspended in 1 ml of sterile normal saline and used immediately to spike the fish fillets.

### Seafood preparation, spiking and growth rate analysis

We have selected common marine fish, Indian Mackerel (*Rastrelliger kanagurta*) of the Indian Ocean to this study. Fresh fish collected from the local market (Cochin) was utilized in the preparation of fillets. Fish fillets of smaller size (~6 x12cm) were prepared by removing skin and gut regions, aseptically and total of 800 g was included in the study. The surface of fish fillets was wiped with ethanol to eliminate background flora and subsequently rinsed with sterile normal saline to remove the impact of ethanol. Fish fillets were spiked with 2x10^7^CFU/400 g of fresh and active culture of *Salmonella* Weltevreden and the inoculum was uniformly distributed over fillets using a sterile cotton swab. Similarly, another batch of fish fillets was spiked with 2x10^6^CFU/400 g active culture of *Salmonella* Typhi and inoculum was distributed uniformly as mentioned above. Both batches of spiked seafood samples were divided the into four different groups and each group (100 g) was incubated, separately at −20 °C in Deep freezer (Vestfrost, India), room temperature (26 ± 1 °C), at 4 °C in BOD incubator (Kemi, India) and at 45 °C incubator (GFL, Germany). Survival count of *Salmonella* Weltevreden and *Salmonella* Typhi was determined at 0, 1, 3, 5, 7 days interval from each group stored at −20, 4, RT and 45 °C on xylose lysine deoxycholate agar and ChROMagar™ *Salmonella* followed by serological confirmation [30]. Unless otherwise stated all dehydrated bacterial culture media were procured from BD, USA.

### RNA extraction and estimation

*Salmonella* Weltevreden and *Salmonella* Typhi samples were drawn for RNA isolation at 0, 1, 3, 5, 7 days interval from individual temperature group stored at −20, 4, RT and 45 °C. Roughly 2g of fish fillets was mixed by vortexing with1 ml of sterile H_2_O and subjected to low centrifugation at 500 × *g* for 2 min to settle down the seafood debris. The pellet was used for isolation of total RNA. For frozen fillets (−20 °C), a small porti on was thawed each time to withdraw the sample and rest of steps followed for RNA isolation were same as in case of other samples. RNA extraction from bacterial cells was performed with RNeasy Protect Bacterial Mini Kit (Qiagen, India) following the manufacturer’s instructions for Gram-negative bacteria. Contamination of the genomic DNA from each RNA preparation was removed using the Turbo DNA-free™ (Ambion, Life Technologies, USA), according to the manufacturer’s instruction for rigorous DNase treatment. Quantification of the total RNA was determined using Qubit® (Life Technologies, USA) and the quality of RNA was determined using Bioanalyzer 2100 (Agilent Technologies, USA). Total RNA isolated from samples was immediately taken for cDNA synthesis.

### cDNA synthesis and relative expression

*Salmonella Salmonella* Weltevreden and Typhi stress (*rpo*E) and virulence genes (*fim*A, *stn*, *inv*A) in fish fillets at −20, 4, RT and 45 °C was determined using real-time PCR based differential gene expression study. Relative expression by qRT-pCR used *gapdh* as an endogenous reference gene in this study. The sequences for all primers used in this study were designed from accession number NC_003197 using DNASTAR Inc. (USA) and primers are listed in Table 1. cDNA was synthesized using Express One-Step qRT-PCR SYBR Green synthesis kit (Invitrogen, Life Technologies, USA) with specific primers as per manufacturer’s instructions. qRT-PCR assay was carried out in Chromo4™ DNA Engine (Bio-Rad, USA) real- time system. The reaction constituents consisted of Express SYBR GreenER supermix, 0.2 uM of each primers, ~250 ng of total RNA and final volume of reaction was made upto 20 μl. The cycling conditions were 50 °C for 5 min (cDNA synthesis), 95 °C for 2 mi n followed by 40 cycles of 95 °C for 15 s and 60 °C for 1 min. Subsequently melting curve analysis was performed between 60 and 95 °C at a transition rate of 0.1 °C/s to confirm the specificity of the PCR products. Each set of experiment was included with No Reverse Transcriptase Control (NRTC) to confirm the absence of genomic DNA contamination. Relative expression was calculated based on 2^-∆∆C^_T_ equation [31].

**Table 1:** PCR primers used in qRT- PCR gene expression assays

Gene	Product size (bp)	Sequence (5’-3’)
*fimA*	92	TGTGCCGTCAGCACTAAATCTGGTGTTATCTGCCTGACCA
*invA*	268	GTGAAATTATCGCCACGTTCGGGCAATCATCGCACCGTCAAAGGAACC
*Stn*	181	TGTGCCGTCAGCACTAAATCTGGTGTTATCTGCCTGACCA
*rpoE*	165	GGTAGTTCTTCGCGGTATTGACATAAAGTGGCGAGTCTGGTTTC
*gapdh*	215	ACCGTTGAAATCGGTAGATACAATAGGTAAAGTACTGCCGGAACTG

### Statistical analysis

The effect of storage at −20, 4, RT and 45 °C on growth of cells, stress and virulence gene expression was investigated in replicates by three independent experiments. Real-time PCR assay was conducted in duplicate and data was analyzed using ANOVA.
